# Circulating soluble receptor for advanced glycation end product: Cross-sectional associations with cardiac markers and subclinical vascular disease in older men with and without diabetes

**DOI:** 10.1016/j.atherosclerosis.2017.07.008

**Published:** 2017-09

**Authors:** S. Goya Wannamethee, Paul Welsh, Olia Papacosta, Elizabeth A. Ellins, Julian P.J. Halcox, Peter H. Whincup, Naveed Sattar

**Affiliations:** a UCL Department of Primary Care & Population Health, UCL Medical School, Rowland Hill Street, London, NW3 2PF, UK; b Institute of Cardiovascular & Medical Sciences, BHF Glasgow Cardiovascular Research Centre, University of Glasgow, Glasgow, UK; c Institute of Life Sciences, Swansea University, Singleton Park, Swansea, SA2 8PP, UK; d Population Health Research Institute, St George's, University of London, Cranmer Terrace, London, SW17 0RE, UK

**Keywords:** Soluble receptor advanced glycation end products, Arterial stiffness, Arterial wave reflections

## Abstract

**Background and aims:**

The soluble receptor for advanced glycation end products (sRAGE) has been implicated in diabetic vascular complications. We have examined the association between sRAGE and cardiac markers [NT-proBNP and cardiac troponin T (cTnT)] and subclinical vascular markers in older men with and without diabetes.

**Methods:**

We performed a cross-sectional study of 1159 men aged 71–92 years with no history of cardiovascular disease (myocardial infarction, stroke, heart failure, coronary artery bypass graft operation or angioplasty). Prevalent diabetes included men with a doctor diagnosis of diabetes, men with fasting glucose ≥7 mmol/l or HbA1c ≥ 6.5% (N = 180). Subclinical vascular measurements included carotid intima media thickness (cIMT), arterial stiffness [pulse wave velocity (PWV)], central aortic blood pressure and arterial wave reflections [central augmentation pressure (AP) and augmentation index (AIx)].

**Results:**

sRAGE was strongly and positively associated with renal dysfunction in men with and without diabetes. sRAGE was significantly and positively associated with NT-proBNP (but not cTnT) and AP and AIx in both groups of men after adjustment for CVD risk and metabolic risk markers, renal function and inflammation. However, no association was seen between sRAGE and central aortic blood pressure, cIMT or arterial stiffness as determined by PWV in either group.

**Conclusions:**

Higher plasma sRAGE was associated with increased NT-proBNP and markers of arterial wave reflections in men both with and without diabetes. Increased sRAGE may contribute to or be a marker of worsening cardiac dysfunction or HF. Further studies with cardiac imaging data are required to confirm this.

## Highlights

• In men with no diagnosed CVD, sRAGE is associated with the cardiac marker NT-proBNP.
• sRAGE also related positively to markers of arterial wave reflection.
• These associations were present in both men with and without diabetes.
• Whether sRAGE predicts worsening cardiac dysfunction and heart failure needs testing.

## 1 Introduction

Advanced glycation end products (AGEs) are bioactive molecules found in high amounts in the western diet, which have been implicated in the pathogenesis of atherosclerosis and heart failure (HF), particularly in patients with diabetes [1], [2], [3], [4]. Numerous studies highlight the interaction between AGEs with their receptor (RAGE), which is expressed in the vasculature, kidney and inflammatory cells, as a potential contributor to increased oxidative stress and inflammation, vascular endothelial dysfunction and arterial stiffening [1], [2]. Enhanced accumulation of AGEs is not just restricted to diabetes but also occurs with natural aging [5]. AGEs may also contribute to the development of vascular disease in non-diabetic people through their pro-oxidant properties [5], [6], [7].

The soluble receptor for advanced glycation end products (sRAGE) is the isoform of RAGE found in serum and is formed by proteolytic cleavage of RAGE [1]. sRAGE appears to act as a decoy for capturing circulating AGEs, preventing them from binding to the cell surface receptor and protecting them from the pro-inflammatory effect of RAGE signalling [1]. While infusion of AGEs is thought to result in upregulation of cell-bound RAGE expression in cardiac tissue, administration of recombinant sRAGE has been shown to inhibit the development and progression of atherosclerosis in animal models [1]. However, the role of sRAGE in cardiovascular disease is still contentious. Circulating sRAGE is strongly associated with increased circulating levels of AGEs in both people with diabetes and without diabetes, and is shown to reflect tissue RAGE expression [8]. Data predominantly from participants in clinical trials have shown sRAGE to be associated with the development of CVD complications and mortality in patients with type-1 and type-2 diabetes [9], [10], [11], [12]. It is suggested that sRAGE, which is easily measured in plasma and serum, could be used to monitor diabetic vascular disease risk as well as to evaluate the effect of potential intervention with a view to modulating sRAGE [13]. However, other studies, largely in people without diabetes, have shown sRAGE to be inversely associated with CVD [14], [15], [16], [17], and less is known about the clinical significance of sRAGE in the general aging population. Non-invasive vascular markers and central haemodynamic measurements (including aortic PWV, central aortic blood pressure and arterial wave reflection) are important markers of early arterial or vascular damage and provide potential proxy indicators of CVD risk [18], [19], [20], [21], [22]. To address the controversy regarding the possible role of sRAGE in CVD, we have investigated the association between circulating sRAGE and early markers of vascular disease including arterial stiffness [pulse wave velocity (PWV)], central aortic blood pressure, arterial wave reflections [central augmentation pressure (AP), augmentation index (AIx)] and intima-media thickness (IMT), as well as biomarkers of subclinical myocardial injury and stress [cardiac troponin T (cTnT) and N-terminal pro B-type natriuretic peptide (NT-proBNP)], separately in those with and without diabetes. We hypothesised that sRAGE would be associated with myocardial damage/dysfunction and subclinical vascular disease before the development of overt cardiac disease and that these associations may depend on the individual's diabetic status.

## 2 Patients and methods

The British Regional Heart Study is a prospective study, which recruited a socioeconomically and geographically representative cohort of 7735 men from 24 British towns between 1978 and 1980. In 2010–2012, all surviving men (n = 3137), aged between 71 and 92 years, were sent a postal questionnaire and invited for a 30^th^ year re-examination. 2137 (68%) men completed the postal questionnaire and 1722 (55%) men attended the re-examination [23]. Ethical approval has been obtained from all relevant local research ethics committees. Blood samples were collected after fasting for a minimum of 6 h and were stored at −70 °C. The men were asked whether a doctor had ever told them that they had myocardial infarction (heart attack, coronary thrombosis), stroke, heart failure or diabetes and to bring their medication to the examination session. They were also asked if they had been told by their doctor if they had narrowing or hardening of the leg arteries (including claudication) and whether they had ever had a coronary artery bypass graft operation (CABG) or angioplasty (percutaneous coronary intervention).

### 2.1 Cardiovascular risk factors

Anthropometric measurements including body weight and height were carried out. Body mass index (BMI) was calculated as weight/(height)^2^ (kg/m^2^). Details of measurements and classification methods for smoking status, alcohol intake and physical activity have been described [24]. The use of antihypertensive medication was based on self-reported medication history and review of codes from the British National Formulary (BNF). Blood pressure was measured using an Omron blood pressure recorder twice in succession in the right arm, with the subject seated and the arm supported, and the mean of the two blood pressure recordings was used. Plasma glucose was measured by a glucose oxidase method using a Falcor 600 automated analyser. HbA1c was measured in whole blood using high performance liquid chromatography. Serum insulin was measured using an ELISA assay, which does not cross-react with proinsulin. Glucose and insulin concentrations were adjusted for the effects of fasting duration and time of day [25]. Predicted glomerular filtration rate (eGFR) (measure of renal function) was estimated from serum creatinine using the equation eGFR = 186 × (Creatinine/88.4)^−1.154^ x (Age)^−0.203^ [26]. C-reactive protein (CRP) was assayed by ultra-sensitive nephelometry (Dade Behring, Milton Keynes, UK). Plasma levels of IL-6 were measured with ELISA (R&D Systems, Oxford, UK). Prevalent diabetes included men with a doctor-diagnosed diabetes and men with fasting blood glucose ≥7 mmol/l or men with HbA1c ≥ 6.5.

### 2.2 NT-proBNP and cardiac troponin T

NT-proBNP and cTnT were measured in plasma samples on an automated clinically validated immunoassay analyser (e411, Roche Diagnostics, Burgess Hill, United Kingdom) using the manufacturers' calibrators and quality control reagents [27]. The lower limit of sensitivity was 5 pg/ml for NT-proBNP and 3 pg/ml for cTnT. Low control coefficient of variation (CV) was 6.7% and high control CV was 4.9%.

### 2.3 Plasma sRAGE

Plasma sRAGE was measured in samples stored at −80 °C until assay, using a commercially available ELISA (R&D Systems, Oxon, UK). The control intra-assay coefficient of variation (CV) was 2.7%, and inter-assay CV was 9.3%.

### 2.4 Non-invasive cardiovascular markers

Measurements were measured by two vascular technicians in series. Left and right carotid arteries were images using a Z.One Ultra ultrasound system (Zonare Medical Systems, Mountain View, CA) with a 5–10-mHz linear probe. A cross sectional sweep from the base of the common carotid artery to the jaw bone and longitudinal images of the common carotid artery approximately 1 cm proximal to the carotid bifurcation were recorded. Peak systolic and end-diastolic common carotid artery diameter and carotid intima media thickness (CIMT) (the distance between the leading edge of the intima and the media-adventitia interface) were measured using the Carotid Analyser software (Medical Imaging Applications, Iowa City, IA). From the longitudinal images, a region of interest (5–10 mm) was selected in a plaque free area, at least 1 cm from the bifurcation. cIMT was measured from three end-diastolic images on each side and a mean of these measures was calculated. Maximum and minimum carotid artery diameter was assessed from three consecutive waveforms and mean distension was calculated. The distensibility coefficient was then calculated using the following formula, as described by Dijk et al.: distensibility coefficient (DC) = [(2× mean distension/baseline diameter)/mean pulse pressure (kPa)]*1000 [28]. There was good agreement between the vascular technicians in ultrasound-based measurements. With regard to the inter- and intra-observer reproducibility, the coefficient of variation (CV) for cIMT (n = 109) was 7.1% and 5.1% respectively. The corresponding inter and intra-observer reproducibility CV for distension (n = 109) was 9.2% and 11.9%, respectively.

The Vicorder (Skidmore Medical, Bristol UK) was used to assess brachial artery waveforms and carotid to femoral pulse wave velocity (PWV). Brachial artery waveforms were recorded with the participant seated with a Hokanson SC10 cuff positioned around the middle of the right upper arm. A blood pressure measurement was taken first and then the cuff was reinflated to diastolic pressure and, when a good quality stable waveform was achieved, the signal was recorded. Central blood pressure (BP), augmentation pressure (AP) and the augmentation index (AIx) were all derived from the pulse waveform using a brachial-aortic transfer function within the Vicorder software. Two readings of AP within ≤5 mmHg and AIx within ≤5% of each other were accepted and averaged. PWV was assessed with participants in a semi-supine position with their torso at approximately 30°. A 2 × 9 cm cuff was positioned around the neck with the bladder over the right carotid pulse, and a Hokanson SC10 cuff around the middle of the right thigh. Path length was measured from the sternal notch to the centre of the thigh cuff. The cuffs were simultaneously inflated and traces with a minimum of 3 good quality waveforms recorded. Two PWV measurements, within ≤0.5 m/s of each other, were accepted and averaged.

### 2.5 Statistical analysis

Distributions of HbA1c, glucose, insulin, CRP, IL-6, NT-proBNP and cTnT were highly skewed and log transformation was used. The men were divided into equal quartiles based on the sRAGE distribution in all men (<6.65, 6.65–6.94, 6.95–7.23 and >7.24 pg/l). For comparisons of baseline characteristics, logistic regression and linear regression were used to test for trends, fitting sRAGE in its original continuous form. Multiple linear regression models were used to assess the association between sRAGE and cardiac markers and subclinical vascular disease measures. In multivariate analyses, use of antihypertensive treatment was fitted as categorical variables whilst age, body mass index (BMI), eGFR, LDL-C, IL-6, insulin, heart rate and NT-proBNP were fitted as continuous variables. All analyses were performed using SAS version 9.3 (SAS, Cary, North Carolina).

### 2.6 Study sample

This present study is a cross-sectional analysis of the data from the 2010–2012 examination. Of the 1722 men who attended the re-examination, sRAGE measurements were available in 1603 men. Men with prior clinical diagnosis of CVD were defined as men with a self-reported doctor diagnosis of CHD, stroke, heart failure or men who had reported a CABG or angioplasty (n = 444). Patient recall of a doctor diagnosis of CHD has been shown to be a valid measure of recording diseases in this study population [29] and [30]. The kappa statistics comparing health record review with patient's recall of CHD was 0.82 [29]. Men with prevalent CVD showed significantly higher mean sRAGE [mean (SD) 7.03 (0.60)] compared to those without prevalent CVD [mean (SD) 6.94 (0.49)] (*p* = 0.002) and were excluded, leaving 1159 men for analysis.

## 3 Results

**Fig. 1:**
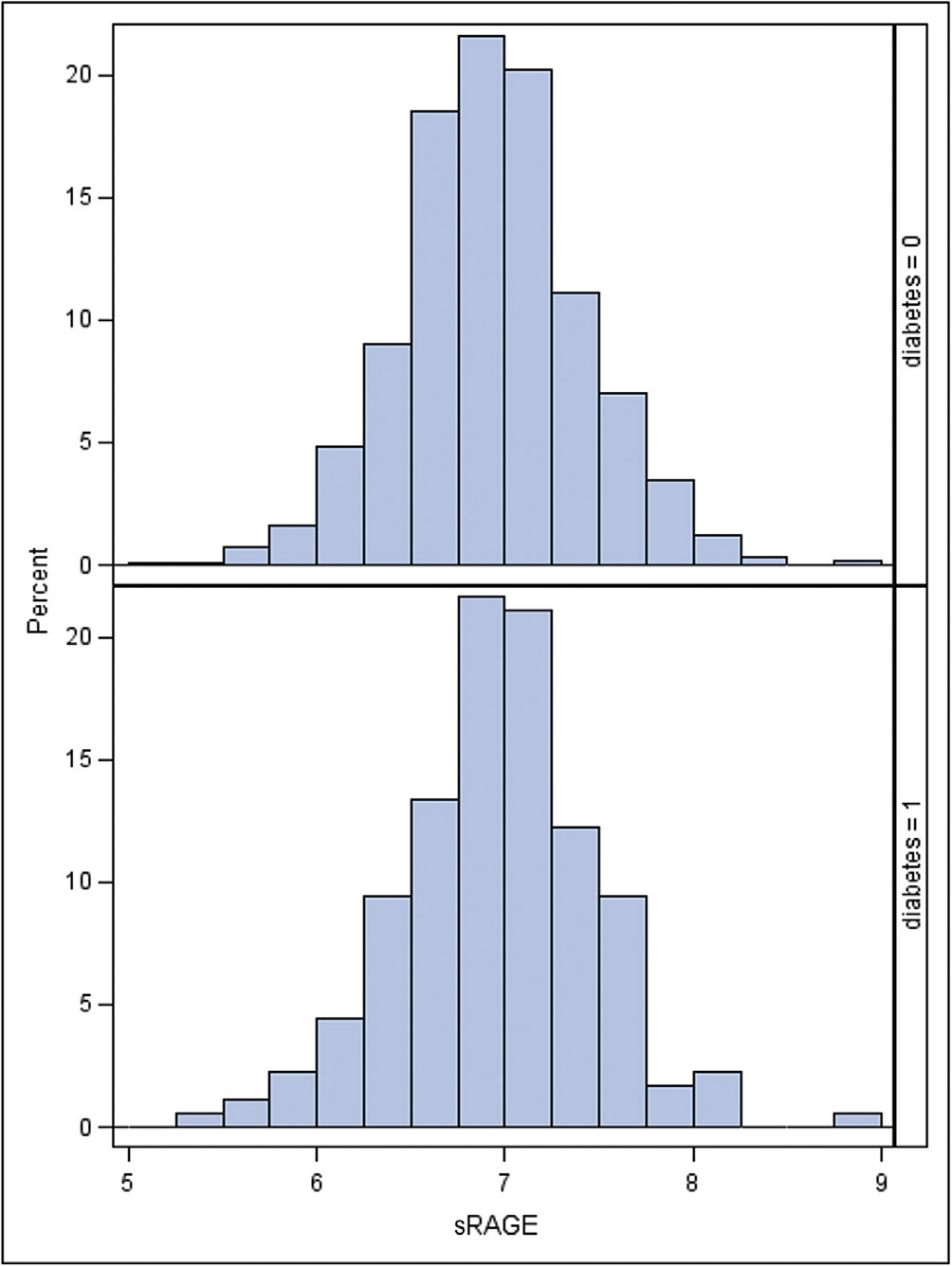
Histogram of the distribution of sRAGE in men with and without diabetes.

In men with no prior clinical diagnosis of CVD, those with diabetes (N = 180) showed similar mean sRAGE (SD) compared to those with no diabetes (N = 979) [6.96 (0.52) *vs.* 6.94 (0.49)]. Fig. 1 shows the distribution of sRAGE in those with and without diabetes. Table 1 shows the baseline characteristics by quartiles of the sRAGE distribution, separately in men without CVD, with and without diabetes. In those without diabetes, sRAGE was positively and significantly associated with age and eGFR (renal dysfunction) but was inversely associated with BMI, blood glucose, insulin, heart rate, and to a lesser extent with IL-6. In men with diabetes, sRAGE also related positively to age and eGFR but weak positive associations were seen with IL-6, and a significant positive association was seen with use of antihypertensive treatment. In contrast to those without diabetes, no association was seen between sRAGE and BMI, glucose, insulin or heart rate.

**Table 1:** Baseline demographic, clinical and metabolic characteristics by quartiles of SRAGE in 1159 men with and without diabetes.

	Men with no diabetes (N = 979) sRAGE (quartiles)				*p*-trend
	1 (lowest) (N = 251)	2 (N = 243)	3 (N = 244)	4 (highest) (N = 241)	
SRAGE (pg/l)	6.34 (0.26)	6.79 (0.09)	7.08 (0.09)	7.56 (0.27)	
Age (years)	77.98 (4.44)	77.87 (4.62)	78.85 (4.84)	79.05 (5.07)	0.0002
BMI (kg/m^2^)	27.21 (3.62)	26.54 (3.80)	26.44 (3.21)	25.84 (3.47)	<0.0001
% smokers	2.4	4.7	4.0	3.2	0.54
% inactive	22.6	20.8	22.1	22.1	0.90
% manual	38.03	46.5	46.3	41.9	0.50
% moderate/heavy drinkers	8.2	5.4	4.2	2.6	0.002
% with PVD^a^	0.8	2.9	3.0	1.3	0.96
Antihypertensive treatment	45.4	42.4	48.0	41.9	0.60
SBP (mmHg)	147.9 (17.5)	149.8 (18.0)	147.6 (18.3)	148.3 (19.1)	0.67
LDL-C (mmol/l)	2.86 (0.89)	2.89 (0.85)	2.93 (0.94)	2.99 (0.99)	0.05
HDL-C (mmol/l)	1.53 (0.44)	1.56 (0.45)	1.46 (0.37)	1.49 (0.45)	0.14
Glucose (mmol/l)^b^	5.37 (4.98–5.76)	5.37 (5.04–5.64)	5.26 (4.95–5.58)	5.26 (4.89–5.53)	0.006
Insulin (IU)^b^	7.46 (5.16–10.92)	7.69 (5.25–11.50)	7.39 (5.11–10.70)	6.04 (4.63–9.52)	0.04
eGFR (ml/min/1.73 m^2^)	78.76 (15.95)	76.36 (15.81)	73.08 (16.93)	68.79 (17.59)	<0.0001
CRP (mg/l)^b^	1.57 (0.65–3.36)	1.30 (0.52–2.78)	1.30 (0.71–2.62)	1.30 (0.67–2.50)	0.15
IL-6 (ng/ml)^b^	3.25 (1.97–4.64)	2.80 (1.75–4.23)	2.89 (1.73–4.18)	2.77 (1.74–4.03)	0.10
Heart rate (beats/min)	70.91 (14.35)	67.27 (12.42)	66.47 (12.58)	66.87 (12.02)	0.01
	Men with diabetes (N = 180)				

1 (N = 46)	2 (N = 42)	3 (N = 45)	4 (N = 47)

sRAGE (pg/l)	6.32 (0.30)	6.83 (0.08)	7.08 (0.08)	7.58 (0.29)	
Age (years)	77.38 (3.79)	78.10 (4.74)	78.86 (4.13)	78.92 (4.76)	0.03
BMI (kg/m^2^)	28.86 (3.43)	28.69 (3.69)	27.97 (4.51)	29.83 (4.87)	0.99
% smokers	4.4	2.1	4.3	4.2	0.35
% inactive	29.6	11.6	26.1	23.3	0.39
% manual	60.9	52.4	48.9	66.0	0.63
% moderate/heavy drinkers	4.4	2.6	6.8	6.5	0.40
% with diagnosed PVD^a^	2.3	0	2.3	4.6	0.62
Antihypertensive treatment	56.5	57.1	73.3	70.2	0.05
SBP	145.2 (20.8)	144.5 (20.9)	140.9 (18.7)	143.5 (22.3)	0.95
LDL-C (mmol/l)	2.46 (0.86)	1.90 (0.85)	2.03 (0.81)	2.01 (0.59)	0.03
HDL-C (mmol/l)	1.35 (0.39)	1.28 (0.36)	1.40 (0.52)	1.22 (0.37)	0.81
Glucose (mmol/l)^b^	7.24 (5.99–8.06)	7.02 (6.01–7.85)	7.00 (6.15–8.03)	7.39 (5.82–9.02)	0.51
Insulin (IU)^b^	10.48 (6.08–18.00)	9.03 (6.06–13.20)	14.43 (9.30–22.84)	14.30 (10.20–21.19)	0.44
eGFR (ml/min/1.73 m^2^)	78.31 (14.83)	75.56 (17.98)	71.53 (16.93)	57.42 (17.72)	<0.0001
CRP (mg/l)^b^	1.75 (0.85–3.58)	1.01 (0.64–1.80)	1.16 (0.49–2.48)	1.63 (0.65–4.59)	0.55
IL-6 (ng/ml)^b^	3.00 (1.88–4.77)	3.00 (1.94–5.21)	2.83 (1.65–4.85)	3.97 (2.39–7.19)	0.30
Heart rate (beats/min)	67.76 (11.16)	65.95 (13.65)	67.78 (11.23)	70.15 (14.79)	0.29

Mean and standard deviation unless specified.

a Peripheral vascular disease defined as self-reported doctor diagnosis of narrowing or hardening of the leg arteries (including claudication).

b Geometric mean (interquartile range).

### 3.1 sRAGE and vascular measurements

**Fig. 2:**
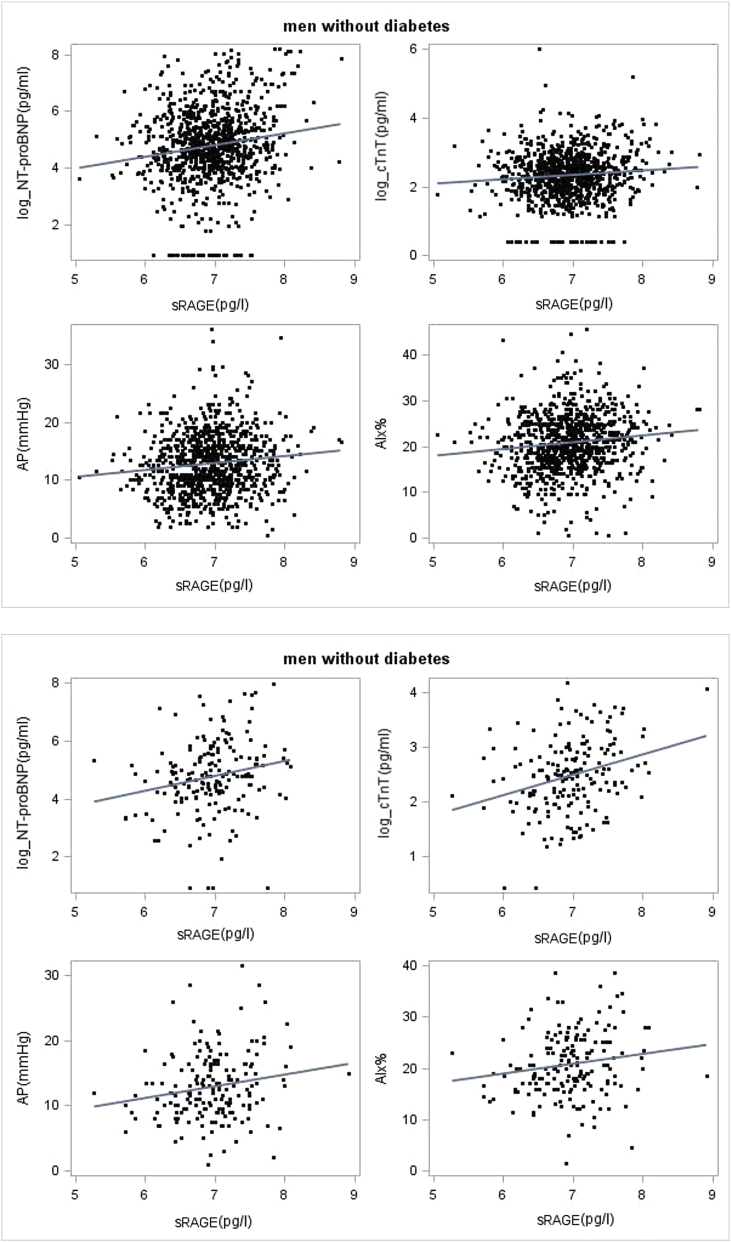
Scatterplot and linear regression line of sRAGE and log NT-proBNP, log cTnT, AP and AIx in men without diabetes and with diabetes. Men without diabetes: correlation coefficient (r) between sRAGE and (i) NT-proBNP (r = 0.15), (ii) AP (r = 0.11), (iii) AIx (r = 0.12) and (iv) cTnT (r = 0.09). Men with diabetes: correlation coefficient (r) between sRAGE and (i) NT-proBNP (r = 0.26), (ii) AP (r = 0.18), (iii) AIx (r = 0.16) and (iv) cTnT (r = 0.29).

sRAGE was positively and significantly associated with the cardiac markers NT-proBNP and cTnT and the haemodynamic parameters central AP and AIx in both men with and without diabetes (Table 2). A weak positive association was seen with aortic central BP. No association was seen with aortic or carotid stiffness (PWV and distensibility respectively) or cIMT in either group. Fig. 2 shows the correlation and linear regression line between sRAGE and NT-proBNP, cTnT, central AP and AIx. The association between sRAGE and NT-pro-BNP (but not cTnT), AP and AIx remained after adjustment for factors shown to be associated with sRAGE in Table 1, and known to be associated with CVD including age, BMI, eGFR, antihypertensive treatment, LDL-c, insulin and IL-6. Results were similar if glucose was used instead of insulin. The adjusted β regression coefficients for sRAGE are presented in Table 3. Further adjustment for heart rate made only minor differences to the associations. The association between sRAGE and AP and AIx was attenuated, but remained significant after further adjustment for NT-proBNP (Table 3). In a sensitivity analysis, we further excluded 23 men who reported claudication. This made little difference to the findings.

**Table 2:** Cardiac markers and subclinical vascular measurements by quartiles of SRAGE in men with and without diabetes.

	sRAGE (quartiles)				*p*-trend
	1 (lowest)	2	3	4 (highest)	
**Men with no diabetes**					
NT-proBNP (pg/ml)^a^	104.6 (54–240)	93.7 (57–187)	129.0 (67–247)	152.9 (68–345)	<0.0001
cTnT (pg/ml)^a^	10.17 (6.57–15.3)	9.39 (6.83–13.41)	10.28 (7.17–15.32)	11.13 (7.26–16.74)	0.006
Central aortic blood pressure (mmHg)	59.19 (12.59)	60.94 (12.21)	61.37 (13.91)	61.21 (13.56)	0.13
Central augmentation pressure (mmHg)	11.47 (4.72)	12.89 (4.60)	13.32 (3.36)	13.29 (5.32)	0.0005
Augmentation index (%)	18.99 (6.05)	21.04 (5.91)	21.42 (6.13)	21.35 (6.36)	0.0003
PWV (m/s)	10.36 (1.62)	10.04 (1.78)	10.14 (1.62)	10.35 (1.82)	0.21
DC (10^−3^ kPa^−1^)	12.02 (3.96)	12.47 (4.52)	11.96 (4.00)	12.37 (4.22)	0.66
cIMT (mm)	0.80 (0.16)	0.80 (0.15)	0.81 (0.16)	0.80 (0.15)	0.58
**Men with diabetes**					
NT-proBNP^a^	79.0 (50–140)	111 (64–235)	131.6 (71–343)	164.0 (76–282)	0.006
cTnT (pg/ml)^a^	8.94 (6.38–13.10)	12.30 (8.19–18.52)	11.25 (7.31–18.00)	15.64 (10.36–26.84)	0.0002
Central aortic blood pressure (mmHg)	59.93 (13.27)	62.14 (13.57)	59.80 (13.98)	64.44 (12.72)	0.25
Central augmentation pressure (mmHg)	11.58 (4.71)	13.46 (4.91)	12.16 (4.18)	14.20 (6.36)	0.02
Augmentation index (%)	19.31 (5.52)	21.50 (6.89)	20.16 (4.94)	22.03 (7.51)	0.04
PWV (m/s)	10.40 (1.53)	10.39 (1.57)	10.02 (1.43)	10.25 (1.20)	0.31
DC (10^−3^ kPa^−1^)	12.50 (4.19)	12.68 (3.38)	11.99 (3.85)	12.49 (4.70)	0.80
cIMT (mm)	0.83 (0.22)	0.80 (0.14)	0.79 (0.14)	0.83 (0.18)	0.43

Mean and standard deviation unless specified.

a Geometric mean (interquartile range).

**Table 3:** Multivariate linear regression models of the relations of SRAGE with cardiac markers and subclinical vascular measurements in men with and without diabetes.

	Age-adjusted β-coefficient (se)	*p*-value	Model 1^a^ β-coefficient (se)	*p*-value	Model 2^b^ β-coefficient (se)	*p*-value	Model 3^c^ β-coefficient (se)	*p-*value
**Men with no diabetes**								
Log NT-proBNP	0.32 (0.08)	0.0002	0.30 (0.09)	0.0007	0.29 (0.09)	0.001		
Log cTnT	0.05 (0.04)	0.20	0.04 (0.04)	0.32	0.04 (0.04)	0.35		
Augmentation pressure	0.99 (0.33)	0.003	1.08 (0.35)	0.002	0.75 (0.33)	0.02	0.70 (0.33)	0.04
Augmentation index	1.37 (0.41)	0.0008	1.52 (0.43)	0.0004	1.14 (0.40)	0.005	1.09 (0.40)	0.008

**Men with diabetes**								
Log NT-proBNP	0.53 (0.18)	0.003	0.42 (0.20)	0.04	0.41 (0.20)	0.04		
Log cTnT	0.31 (0.09)	0.0007	0.14 (0.09)	0.13	0.14 (0.09)	0.12		
Augmentation pressure	1.61 (0.75)	0.03	1.42 (0.79)	0.07	1.59 (0.72)	0.03	1.43 (0.72)	0.05
Augmentation index	1.82 (0.92)	0.05	1.98 (0.99)	0.04	2.21 (0.89)	0.01	1.83 (0.89)	0.04

a Model 1:adjusted for age, BMI, eGFR, antihypertensive treatment, LDL-C, insulin and Il-6.

b Model 2:Model 1 + heart rate.

c Model 3:Model 2+NT-proBNP.

## 4 Discussion

In this study of older men without prevalent CVD, sRAGE was strongly and positively associated with renal dysfunction, as observed in other studies [31], and related positively to NT-proBNP, a marker of ventricular stress, as well as haemodynamic markers of arterial wave reflection (AP and AIx), which are known to predict CVD and HF [18], [19], [20], [21], [22], [27]. Interestingly, levels of sRAGE were similar in men with and without diabetes and these associations with NT-proBNP, AP and AIx were seen in elderly men both with and without diabetes. To our knowledge, this is the first study to report on the association between sRAGE and both cardiac and vascular risk markers in those with and without diabetes from within the same population. Our findings of a positive association between sRAGE and both NT-proBNP and haemodynamic parameters known to be associated with CVD risk supports prospective studies that have shown sRAGE to be positively associated with increased risk of incident CVD in diabetes and in older adults and provides further insight into upstream pathways by which sRAGE may influence clinical development of CVD.

### 4.1 sRAGE and CVD

Although a number of cross sectional and prospective studies have shown inverse associations between sRAGE and risk of CVD, several other prospective studies, particularly in diabetes and the elderly, have shown sRAGE to be positively associated with increased risk of CVD [8], [9], [10], [11], [12]. We have also shown sRAGE to be significantly elevated in those with prevalent CVD, which is consistent with other reports of positive associations between sRAGE and CVD in diabetes and in older adults [8], [9], [10], [11], [12]. It is suggested that in states of increased AGEs, such as in people with diabetes and in older populations, RAGE expression is increased [8]. Thus, sRAGE in diabetes or in older adults may reflect the activity of the AGE-RAGE axis [10].

### 4.2 sRAGE, subclinical arterial disease and central haemodynamics

No association was seen between sRAGE and carotid atherosclerosis, which is consistent with findings from the NOMAS study of older adults [32]. We also found no association between sRAGE and arterial stiffness, as measured by carotid to femoral PWV and carotid distensibility, and only a weak positive association with central aortic pulse pressure. Previous work examining the relationship between sRAGE and arterial stiffness has been inconsistent, with some studies showing positive associations [33] and others finding inverse associations [34]. By contrast, a significant association was seen between sRAGE and AP and AIx, even after adjustment for CVD risk factors including BMI, renal function, metabolic risk markers, inflammation and heart rate. The association between sRAGE and both AP and AIx was partially accounted for by its relationship with NT-proBNP. Although AP and AIx have been used as indirect clinical indices of arterial stiffness, they are also importantly influenced by the timing and magnitude of wave reflections and are considered important measures of wave reflections [35]. Central AIx has also been shown to be affected by changes in LV ejection time and systolic loading [36]. The AIx, which may represent an increase in wave reflections and also serve as a marker of LV afterload, has been associated with impaired left ventricular systolic and diastolic function [37], [38], which may herald the development of clinically overt heart failure. Data on the relationship between sRAGE and AP and AIx are limited and further studies are needed to confirm these findings and their clinical significance.

### 4.3 sRAGE and cardiac function

We have observed a positive association between sRAGE and NT-proBNP, a marker of left ventricular stress, but not with cTnT, a marker of myocardial damage. NT-proBNP is a cardiac hormone secreted from myocytes in response to ventricular and arterial wall stress and is predictive of CVD and HF [27]; circulating levels of NT-proBNP are increased in both LV systolic and diastolic dysfunction. Mechanical stress on cardiac myocytes stimulates secretion of NT-proBNP, and central haemodynamic indices may, at least in part, indicate left ventricular loading conditions. Several lines of evidence suggest that AGEs are related to the development and progression of HF in both diabetic and non-diabetics patients [2]. Clinical studies in animals have also found that compliance of the left ventricle decreases with accumulation of AGEs in the myocardium and implicated AGEs in the development of both diastolic and systolic heart failure. The positive association we found between sRAGE and AP, AIx and NT-proBNP in men both with and without diabetes is consistent with these assertions and may reflect a direct effect of the AGE-RAGE system on left ventricular loading and function. This may explain why sRAGE is associated with the AP/AIx and NT-proBNP, but not large artery stiffness as characterised by aortic PWV, distensibility or cIMT in both men with and without diabetes in our study. Individuals with diabetes are at particularly high risk of CVD and HF [39] and RAGE may be a mechanism by which diabetes may lead to left ventricular dysfunction and eventually HF. Although sRAGE has been shown to be elevated in patients with HF [40], studies on sRAGE and incident HF risk are sparse, with only one prospective study conducted in middle-aged adults showing an inverse association between sRAGE and incident HF [41]. It is possible that sRAGE has differing relationships in differing states of increased AGEs; for example sRAGE in older adults and people with diabetes may reflect the level of RAGE expression.

### 4.4 Strengths and limitations

The study is a geographically representative sample of older British men and we are not aware of previous studies describing associations between sRAGE and a wide range of cardiovascular risk factors and vascular measurements in a large population-based sample of older people both with and without diabetes in whom AGEs are increased. The issue of survivor bias is inevitable in cohorts of ageing populations, with subjects with diabetes and CVD likely to have died at a higher rate. The results presented in this study are also subject to the sampling bias by the moderate response rate for the clinical examination (55%), which is also likely to have excluded participants in worse health; men who participated in our study were healthier than those who did not. These factors may have resulted in some of the null findings seen between sRAGE and vascular measurements such as cIMT and arterial stiffness. Nevertheless, we have observed significant associations between sRAGE and central haemodynamics (arterial wave reflections) and NT-proBNP in men with no prior clinical diagnosis of CVD. sRAGE was based on one measurement, however, concentrations of sRAGE within individuals have shown to be relatively stable over three years [42]. Another limitation of our study is that it comprises only white European men, and the generalizability of the study to women and other ethnic groups is limited. Our findings are based on associations with biological markers associated with cardiac dysfunction. Cardiac imaging measurements were not available and we do not have any direct data on LV systolic/diastolic function or ventricular-arterial interaction. We also acknowledge that our results are based on cross-sectional analyses, therefore, we cannot infer the direction of causation in the associations observed. Our study cannot address the question of whether sRAGE would be a useful clinical tool to assess CVD or HF risk based on cross sectional findings. Nevertheless, the association with wave reflection and cardiac dysfunction (NT-proBNP) may help towards understanding mechanisms and pathways by which elevated sRAGE is associated with increased risk of CVD/HF in diabetic patients. Further prospective studies and clinical studies are warranted to investigate the potential clinical usefulness of measuring sRAGE in clinical practice.

### 4.5 Conclusion

Higher plasma sRAGE is associated with increased NT-proBNP and arterial wave reflections (AP and AIx) in both older men with and without diabetes, but not with cIMT or arterial stiffness. These findings suggest that sRAGE as a marker of augmented AGE-RAGE signalling activity may contribute to or be a marker of worsening cardiac dysfunction and HF and thus may be a potential mechanism by which dysglycemia and diabetes lead to increased risk of HF. Cardiac imaging data are required to confirm these findings. Future prospective studies are warranted to investigate the association between sRAGE and development of HF in older adults with and without diabetes.

## Conflict of interest

The authors declared they do not have anything to disclose regarding conflict of interest with respect to this manuscript.

## Financial support

The British Regional Heart Study is a British Heart Foundation (BHF) research group. This work was supported by the British Heart Foundation program grant (RG/13/16/30528) and project grant (PG/09/024). Dr Paul Welsh is supported by BHF fellowship FS/12/62/29889.

## Author contributions

SGW initiated the concept and design of the paper, analysed the data and drafted the manuscript. JPJH, NS, PW and PHW contributed to the interpretation of data. OP contributed to the analysis of the paper. EAE, JPJH and PW contributed to the acquisition of the data. All authors revised it critically for important intellectual content and approved the final version of the manuscript. SGW is the guarantor and takes full responsibility for the work as a whole, including the study design, access to data, and the decision to submit and publish the manuscript.
